# Supplementary material for: Effects of Silica Shell Encapsulated Nanocrystals on Active χ-Fe5C2 Phase and Fischer–Tropsch Synthesis
Source: Nanomaterials (Basel). 2022 Oct 21;12(20):3704. doi: 10.3390/nano12203704 (PMC9610965; doi:10.3390/nano12203704)
Supplement: Supplementary file 1 [file nanomaterials-12-03704-s001.zip › nanomaterials-1952689-supplementary.pdf]

Supplementary Information

Effects of silica shell encapsulated nanocrystals on active χ-Fe_5C_2 phase and Fischer-Tropsch synthesis

Seunghee Cha[1], Heewon Kim[1], Hyunkyung Choi[2], Chul Sung Kim[2], and Kyoung-Su Ha[1,*]

[1] *Department of Chemical and Biomolecular Engineering, Sogang University, 35 Baekbeom-Ro, Mapo-Gu, Seoul 04107, Republic of Korea;*
[2] *Department of Physics, Kookmin University, 77 Jeongneung-ro, Seongbuk-gu, Seoul, 02707, Republic of Korea*
* Correspondence: K.-S. Ha; philoseus@sogang.ac.kr; Tel.: +82-2-3274-4859

List of SI

Figure S1. XRD result of χ-Fe_5C_2 nanoparticles.

Figure S2. XPS spectra of Fe 2p for χ-Fe_5C_2 @SiO_2 catalysts.

Figure S3. XRD results of fresh and spent χ-Fe_5C_2 @SiO_2_H catalysts.

Table S1. N_2 physisorption results of fresh and spent χ-Fe_5C_2@SiO_2 catalysts**.**

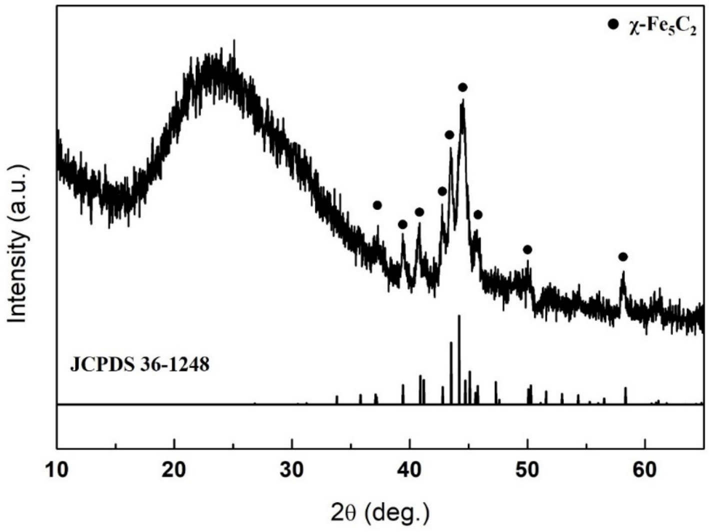

Figure S1. XRD result of χ-Fe_5C_2 nanoparticles.

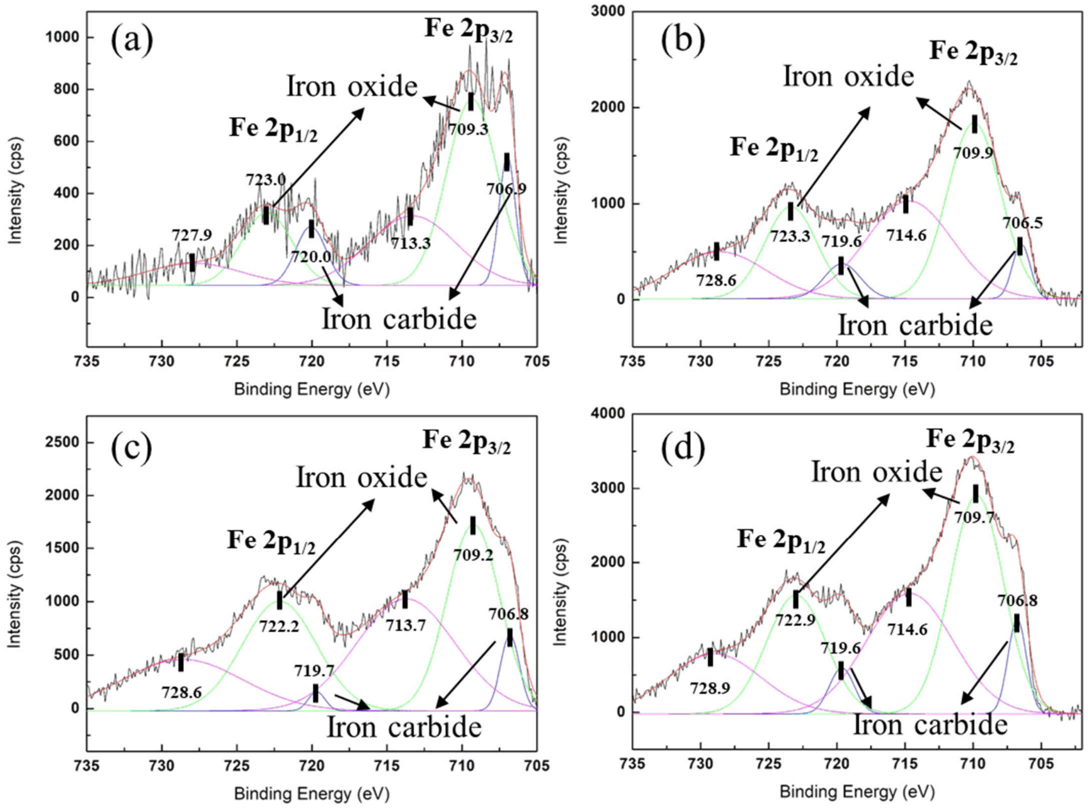

Figure S2. XPS spectra of Fe 2p for (**a**) fresh χ-Fe_5C_2@SiO_2_L catalyst, (**b**) spent χ-Fe_5C_2@SiO_2_L catalyst, (**c**) fresh χ-Fe_5C_2@SiO_2_H catalyst, and (**d**) spent χ-Fe_5C_2@SiO_2_H catalyst after deconvolution.

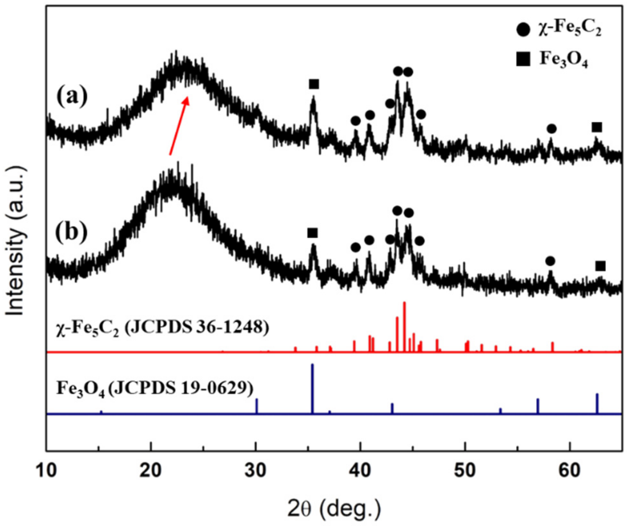

Figure S3. XRD results of fresh and spent χ-Fe_5C_2 @SiO_2_H catalysts. (a) spent catalyst, (b) fresh catalyst.

Table S1. N_2 physisorption results of fresh and spent χ-Fe_5C_2@SiO_2 catalysts**.**

Sample	S_{BET} [m^2/g]	V_p [cm^3/g]	Average d_p [nm]	Mesopore d_p [nm] [a]
Fresh χ-Fe_5C_2@SiO_2_L	301	0.5	7.0	2.3
Spent χ-Fe_5C_2@SiO_2_L	880	1.0	4.1	2.6
Fresh χ-Fe_5C_2@SiO_2_H	238	0.3	5.7	2.2
Spent χ-Fe_5C_2@SiO_2_H	766	0.8	3.9	2.5

[a] Mesopore d_p was calculated with the pores below 10 nm.
